# Prevalence of stress and depression and associated factors among women seeking a first-trimester induced abortion in China: a cross-sectional study

**DOI:** 10.1186/s12978-022-01366-1

**Published:** 2022-03-09

**Authors:** Qiuxiang Zhang, Na Wang, Yinchu Hu, Debra K. Creedy

**Affiliations:** 1 Department of Gynaecology and Obstetrics, Peking University People’s Hospital, 11 Xizhimen Nan Street, Xicheng District, Beijing, 100044 China; 2 School of Nursing, Capital Medical University, 10 Xitoutiao Road, Fengtai District, Beijing, 100069 China; 3 School of Nursing, Peking University, 38 Xueyuan Road, Haidian District, Beijing, 100191 China; 4 School of Nursing and Midwifery, Griffith University Logan Campus, University Drive, Meadowbrook, QLD 4131 Australia

**Keywords:** Abortion, Mental Health, Perceived stress, Depression, Intimate relationship, Domestic violence, Resilience, Social support, China

## Abstract

**Objectives:**

To determine the prevalence of stress and depression and associated factors among women seeking a first-trimester induced abortion in China.

**Methods:**

A cross-sectional study was conducted in a tertiary hospital in Beijing, from April 1st to Oct 31st, 2021. Women seeking termination of an intrauterine first-trimester pregnancy were invited to participate and complete a digital self-administered questionnaire. The survey included socio-demographic and health questions, Perceived Stress Scale-10 (PSS-10), and Patient Health Questionnaire-9 (PHQ-9). Descriptive analyses and binary logistic regression analyses were performed using SPSS 23.0.

**Results:**

A total of 253 women participated. Prevalence of high perceived stress (cut-off ≥ 20) and depressive symptoms (cut-off ≥ 10) was 25.3% and 22.5%, respectively. Women were more likely to suffer high stress if they reported low resilience (aOR = 16.84, 95% CI 5.18–54.79), were not-using contraceptives (aOR = 3.27, 95% CI 1.39–6.29), had low social support (aOR = 2.95, 95% CI 1.39–6.29), were non-local residents (aOR = 2.51, 95% CI 1.15–5.92), were dissatisfied with their intimate relationship (aOR = 2.44, 95% CI 1.15–5.16), or held pro-life attitudes towards abortion (aOR = 1.04, 95% CI 1.18–3.53). Odds of experiencing depression were higher among women who also reported high perceived stress (aOR = 19.00, 95% CI 7.67–47.09), had completed higher education (aOR = 12.28, 95% CI 1.24–121.20), and were non-local residents (aOR = 3.38, 95% CI 1.37–8.32).

**Conclusions:**

The magnitude of perceived stress and depression was high among Chinese women seeking a first-trimester induced abortion. It is necessary to comprehensively evaluate the mental health of women seeking an abortion, especially those with high risk. Interventions to mitigate relevant associated factors could improve the psychological wellbeing of women.

**Supplementary Information:**

The online version contains supplementary material available at 10.1186/s12978-022-01366-1.

## Plain language summary

Abortion is a stressful life event. Understanding how women perceive having an abortion and their psychosocial responses will enable health providers to understand women’s needs and provide high-quality care. This study aims to determine the frequency of stress and depression among women seeking an induced abortion in early pregnancy in China and associated factors Around 25.3% of women reported high stress and 22.5% reported depression. Women were more likely to suffer high stress if they reported low resilience, were not-using contraceptives, had low social support, were non-local residents, were unhappy with their intimate relationship, or held pro-life attitudes towards abortion. High perceived stress, high education level, and non-local status were associated with depression. The findings indicate stress and depression are quite common among women seeking an abortion in China, especially those with high-risk factors. It is necessary to evaluate and promote the mental health of women seeking an abortion.

**Supplementary Information:**

The online version contains supplementary material available at 10.1186/s12978-022-01366-1.

## Background

Around 73 million abortions are performed annually worldwide [1], and more than 90% occur in the first-trimester [2]. A single first-trimester abortion is physically safe when conducted using WHO-recommended methods by qualified professionals [3]. However, in most cases, an abortion is a stressful life event, that happens in the context of an unwanted/unintended pregnancy and poses challenges to women psychological wellbeing [4]. While some women cope well, others suffer a range of mental health consequences [4, 5].

Negative associations between perceived stress and psychological health have been observed across a wide variety of studies conducted among women having a preterm birth [8] and those experiencing a miscarriage [9]. Understanding women’s perceptions of coping with abortion, as well as associated factors, can be helpful for health service providers to assess and plan for individual variation in coping. Various models attempt to explain variability in a person’s response to a stressful life event. The Transactional Model derived from stress and coping theories [4, 6], for example, can help to explain factors affecting women’s response to an abortion experience. According to this model, a woman’s psychological experience is shaped by how she appraises the significance of the abortion and her ability to cope [7]. Perceived stress emerges from situations that women appraise as exceeding their ability or resources to cope [7]. Women’s perceptions of stress can influence their choice of specific coping behaviours and subsequent psychological well-being [7].

Few studies, however, have reported perceptions of stress among women seeking an abortion. Using the 4-item Perceived Stress Scale (PSS-4), Harris et al. [10] revealed minimal stress among 254 women seeking first-trimester abortions in the U.S. (mean of 4.2 on a 0–16 scale). Another study also conducted in the U.S. reported an average PSS-4 score of 7.24 among 784 women at the time of abortion seeking but placed no limitation on gestational age [11]. Variability in findings may be related to heterogeneity in women's gestation and reliability of the PSS-4. A systematic review comparing psychometric properties (e.g. internal consistency reliability, factorial validity, and hypothesis validity) of all three versions of the PSS (4, 10, and 14 items) recommended the PSS-10 to measure perceived stress [12]. Yet, the PSS-10 is rarely used to explore individuals’ perceptions of stress associated with abortion.

Studies on other psychological responses of women to abortion have also reported significantly different outcomes [4]. Prevalence of depression has ranged from 7.9 to 53.5% [13, 14]. In addition to heterogeneity in measures and evaluation timeframe, few studies controlled for possible confounders [5]. Possible confounders were identified by the American Psychological Association (APA) Task Force on Mental Health and Abortion (TFMHA) [4] in a review of 23 studies conducted among U.S. women. Factors such as a prior history of mental health problems, gestational age, low self-esteem, low resilience, low perceived ability to cope with the abortion, ambivalence about the abortion decision, stigma, and low social support were negatively associated women’s abortion psychological experiences.

Another potential factor impacting on women’s psychological outcomes relates to the wider socio-political context within which pregnancy and abortion occur. General statements about the impact of abortion on women psychological wellbeing can be inaccurate, due to the regional differences in legal context, cultural views, as well as associated social stigma of abortion [4, 5]. In 2011, the UK National Collaborating Centre for Mental Health (NCCMH) systematically reviewed a broader international literature on abortion [5]. Among the 27 studies included, only two were conducted in a developing country (South Africa and Russia), with 13 studies conducted in the U.S. and six in Northern Europe [5]. None of the studies had been conducted among the Chinese population.

In China approximately 6–9 million induced abortions are performed every year, corresponding to 15% of the world’s total abortion numbers [15]. China has some of the most liberal abortion laws in the world, which allow first-trimester abortion without restriction as to the reason, with the exception of sex-selective abortions which are prohibited [16]. According to the State of World Population 2018 (UNFPA), many Chinese women report a continued desire for only one child [16], even though the One-Child Policy ended in 2013. Despite legislation that supports women’s reproductive rights and access to safe abortion services, little is known about how Chinese women perceive having an abortion and their psychosocial responses at the time of abortion-seeking. Furthermore, there is no published evidence regarding factors associated with individual variation in women’s psychological well-being.

### Objectives

This study aimed to (1) determine the prevalence of high perceived stress and depression among women seeking a first-trimester induced abortion in China; and (2) identify factors associated with high perceived stress and depression to help detect high-risk groups.

## Methods

### Study design and setting

A cross-sectional study was conducted in the Family Planning and Reproductive Health Centre (FPRHC) of a tertiary hospital in Beijing, China. Participants were recruited from the outpatient unit, where abortion-related services are normally provided.

### Participants

Women meeting the following criteria were invited to participate: (1) Chinese citizens or living in China; (2) could speak, read, and write in Chinese; (3) 18 years or older; (4) seeking termination of an intrauterine pregnancy; (5) less than 12 gestational weeks; and (6) certain about their abortion decision which was of their own free will. Women were excluded if they were: (1) presenting for a post-abortion follow-up examination, secondary treatment of an incomplete abortion, or non-abortion related services; (2) had been infected by COVID-19; (3) had a mental health history; or (4) unable to give informed consent (e.g., had severe intellectual disability).

### Sample size determination

To determine prevalence of high perceived stress and depression, samples of 202 and 248, respectively were needed using a single population proportion formula, rate for high perceived stress of p = 24.7% [17] and rate for depression of p = 22.4% [18], 95% CI, 5% margin error, and design effect at 2. Hence, a sample size above 248 was deemed sufficient.

### Survey content and data collection

Demographic information included participants’ age, marital status, education, religion, income, and were local or non-local residents as recorded in the Chinese household registration (*hukou*) system (determining eligibility for publicly-funded services and welfare [19]). Reproductive history included parity, abortion history, and number of living children. Current circumstances were women’s gestational age, whether the pregnancy was intended, reasons for choosing an abortion, attitude toward abortion, parenthood plan, and contraception use.

### Primary outcomes

#### Perceived stress

The Chinese version of the Perceived Stress Scale-10 (PSS-10) [20] relates to how unpredictable, uncontrollable, and overloaded respondents perceive their current situation since first finding out about the pregnancy to now. Scores range from 0 to 40 with a score ≥ 20 considered high perceived stress [18, 21]. In this study, participants were also asked to indicate any other specific sources of stress in this current pregnancy.

#### Depression symptoms

Depression symptoms were measured by the Patient Health Questionnaire-9 (PHQ-9) [22]. The nine items address symptoms associated with experiencing pleasure, feeling down, and self-esteem. Participants rate the severity of their depressive symptoms since pregnancy on a 4-point scale (0 = not at all; 1 = several days; 2 = more than half the days; and 3 = nearly every day). Total scores range from 0 to 27, with a score ≥ 10 indicating depression. The tool has been translated and valid for use among the Chinese population [23].

### Potential associated factors

#### Domestic violence

Terms such as ‘domestic violence’, ‘intimate partner violence’, and ‘violence against women’ are used interchangeably in the literature. We used the Domestic Violence Screening Tool (DVST) recommended by the American College of Obstetricians and Gynecologists to detect women’s experience of violence during the preceding year by an intimate partner [24]. Three questions relate to physical, mental or verbal, and sexual abuse within the past year. Any positive response indicates the presence of violence. The tool has been translated and is valid for use among Chinese women [25].

#### Intimate relationship satisfaction

The 4-item Couples Satisfaction Index CSI-4 [26] measures intimate relationship quality. The CSI-4 uses different response formats, with total scores ranging from 0 to 21, with higher scores indicating greater satisfaction and scores below 13.5 suggesting notable relationship dissatisfaction. The Cronbach’s α reliability of the translated version with Chinese woman is 0.88 [27].

#### Perceived social support

The Medical Outcomes Study Social Support Scale (MOSSS-5) [28] measured participants’ perceptions of social support since their pregnancy. Each item is scored on a 5-point Likert scale and summed for a total score ranging from 5 to 25. A total score between 5 and 16 is deemed as low perceived social support. The MOSSS-5 has adequate test–retest reliability (0.89) and internal consistency (0.97) in Chinese population [29].

#### Resilience

The Chinese version of the Brief Resilience Scale (BRS) [30], includes six statements about one’s self-efficacy in dealing with stressful events on a scale of 1 = strongly disagree to 5 = strongly agree. Possible mean scores of all six-items are interpreted as low (1–2.99), normal (3–4.30), and high resilience (4.31–5) [31]. The Chinese version BRS has good reliability and internal consistency [32].

### Data collection

A digital self-administered questionnaire was used to collect data. Two nurses employed by the center screened all women based on inclusion and exclusion criteria. After informed consent, eligible women completed the questionnaire on a tablet computer offered by the research team or on their own tablet by scanning a QR code. All participants were offered an optional private room as well as assistance from the first author to complete the questionnaire. Clinic nurses and the first author were trained for scientific research data collection.

### Data analysis

All data were analyzed using Statistical Package for the Social Sciences (SPSS)^®^ version 23 [33]. Descriptive statistics were presented using frequency counts and percentages (categorical variables) or mean ± standard deviation (continuous variables). Relationships between categorized outcomes and explanatory variables were tested by Chi-square. Multivariate binary logistic regression analysis was performed with stress and depression as the dichotomous variables, respectively. Factors with p-value < 0.1 were included in the binary logistic regression model, and p-values < 0.05 were considered significant.

## Results

### Characteristics of participants

In total, 253 eligible women finished the survey with a response rate of 81.3% (253/311). The mean age of participants was 32.08 ± 6.09, with a predominant age group of 25–34 (43.5%) (as shown in Table 1). Nearly two-thirds (64.4%) were married, and half lived locally. Most women (99.2%) were atheists, and 89.7% held pro-choice attitudes toward abortion. Around half (52.6%) had children, with 45.5% having had an abortion before. The average parity was 2.13 ± 0.85 pregnancies. Most (82.2%) participants were less than nine weeks pregnant, with an average gestational age of 7.32 ± 1.30 weeks. Most women (87%) indicated their pregnancy as unintended, and social factors were the most common cited reasons (63.2%) for choosing an abortion. Only seven (2.8%) women reported any type of IPV experience within the last year. However, 31.5% indicated notable intimate relationship dissatisfaction (CSI-4 < 13.5). The mean score of perceived social support was 18.28 ± 5.02, with over a third (35.2%) reporting low support (MOSSS-5 < 17). Participants reported a mean resilience score of 3.56 ± 0.57 with 9.5% reporting low resilience (BRS < 3).

**Table 1:** Demographic and reproductive characteristics of low and high perceived stress participants

Variables	Total n (%) N = 253	Low perceived stress (0–19) n (%) N = 189	High perceived stress (20–40) n (%) N = 64	F/χ^2^ (p value)
Demographic information				
Age				3.12 (0.210)
≤ 24	37 (14.6)	24 (64.9)	13 (35.1)	
25–34	110 (43.5)	81 (73.6)	29 (26.4)	
≥ 35	106 (41.9)	84 (79.2)	22 (20.8)	
Marital status				1.64 (0.201)
Unmarried	90 (35.6)	63 (70.0)	27 (30.0)	
Legally married	163 (64.4)	126 (77.3)	37 (22.7)	
Education				1.05 (0.307)
≤ 12 years	24 (9.5)	20 (83.3)	4 (16.7)	
> 12 years	229 (90.5)	169 (73.8)	60 (26.2)	
Religion				–
Atheist	251 (99.2)	187 (74.5)	64 (25.5)	
Others	2 (0.8)	2 (100.0)	0 (0)	
Monthly income (RMB)				0.51 (0.773)
< 1000	10 (4.0)	7(70.0)	3 (30.0)	
1000–10,000	123 (48.6)	90 (73.2)	33 (26.8)	
> 10,000	120 (47.4)	92 (76.7)	28 (23.3)	
Household registration				**8.12 (0.004)**
Local	126 (49.8)	104 (82.5)	22 (17.5)	
Non-local	127 (50.2)	84 (66.9)	42 (33.1)	
Pre-existing physical condition				0.12 (0.694)
No	245 (96.8)	184 (75.1)	61 (24.6)	
Yes	8 (3.2)	5 (62.5)	3 (37.5)	
Reproductive history				
Parity				**9.44 (0.009)**
1	77 (30.4)	48 (62.3)	29 (37.7)	
2	65 (25.7)	54 (83.1)	11 (16.9)	
≥ 3	111 (43.9)	87 (78.4)	24 (21.6)	
Previous abortion				0.81 (0.369)
No	138 (54.5)	100 (72.5)	38 (27.5)	
Yes	115 (45.5)	89 (77.4)	26 (22.6)	
Existing child (ren)				**6.27 (0.012)**
No	120 (47.4)	81 (67.5)	39 (32.5)	
Yes	133 (52.6)	108 (81.2)	25 (18.8)	
Current status				
Gestation weeks				0.27 (0.601)
Mean	7.32 ± 1.30			
< 9 weeks	208 (82.2)	154 (74.0)	54 (26.0)	
9–12 weeks	45 (17.8)	35 (77.8)	10 (22.2)	
Pregnancy intention				0.08 (0.779)
Unintended	220 (87.0)	165 (75.0)	55 (25.0)	
Intended	33 (13.0)	24 (72.7)	9 (27.3)	
Abortion reason				1.06 (0.590)
Health-related	93 (36.8)	68 (73.1)	25 (26.9)	
Social-related	142 (56.1)	109 (76.8)	33 (23.2)	
Both	18 (7.1)	12 (66.7)	6 (33.2)	
Opinion about abortion				**6.67 (0.010)**
Pro-choice	227 (89.7)	175 (77.1)	52 (22.9)	
Pro-life	26 (10.3)	14 (53.8)	12 (46.2)	
Parenthood plan				3.47 (0.176)
Yes	35 (13.8)	24 (68.6)	11 (31.4)	
Current no, but yes in 2 years	59 (23.3)	40 (67.8)	19 (32.2)	
No	159 (62.8)	125 (78.6)	34 (21.4)	
Contraception use				**5.02 (0.025)**
Yes	209 (82.6)	162 (77.5)	47 (22.5)	
No	44 (17.4)	27 (61.4)	17 (38.6)	
Domestic violence experience				0.00 (1.000)
No	246 (97.2)	184 (74.8)	62 (25.2)	
Yes	7 (2.8)	5 (71.4)	2 (28.6)	
Low resilience				**34.66 (0.001)**
Yes	24 (9.5)	6 (25.0)	18 (75.0)	
No	229 (90.5)	183 (79.9)	46 (20.1)	
Low social support				**28.05 (0.001)**
Yes	89 (35.2)	49 (55.1)	40 (44.9)	
No	164 (64.8)	140 (85.4)	24 (14.6)	
Notable intimate relationship dissatisfaction				**14.64 (0.001)**
Yes < 13.5	92 (36.4)	56 (60.9)	36 (39.1)	
No	161 (63.6)	133 (82.6)	28 (17.4)	

Note: Bold means *p* < 0.05

### Perceived stress and associated factors

Of the 253 responses, the mean perceived stress score was 16.96 ± 0.44, with an overall prevalence of high perceived stress (PSS ≥ 20) at 25.3%. Table 1 presents associations between participants’ characteristics and perceived stress levels when seeking a first-trimester abortion. As shown in Table 2, the logistic regression model revealed that women who were non-local residents, had pro-life attitudes towards abortion, did not use contraception, and had low resilience, low perceived social support, and relationship dissatisfaction were more likely to report high perceived stress. Women’s resilience played the greatest role in perceived stress. The odds ratio of women with low resilience compared to those with high perceived stress was aOR = 16.84 (95% CI 5.18–54.79, p < 0.001).

**Table 2:** Factors associated with high perceived stress (PSS ≥ 20) from binary logistic regression analysis

Variable	High perceived stress n (%)	B-coefficient	p value	aOR	95% CI
Household registration					
Local	22 (17.5)			1.00	
Non-local	42 (**33.1**)	**0.918**	**0.020**	**2.51**	**1.15–5.92**
Parity			0.318		
1	29 (37.7)			1.00	
2	11 (16.9)	0.668	0.286	1.95	0.57–6.65
≥ 3	24 (21.6)	− 0.155	0.775	0.86	0.29–2.49
Child					
No	25 (32.5)			1.00	
Yes	39 (18.8)	− 0.273	0.620	0.76	0.26–2.24
Attitude toward abortion					
Pro-choice	52 (22.9)			1.00	
Pro-life	12 (**46.2**)	**0.714**	**0.010**	**2.04**	1.18–3.53
Contraception use					
Yes	47 (22.5)			1.00	
No	17 (**38.6**)	**1.185**	**0.007**	**3.27**	**1.39–7.69**
Low resilience					
No	46 (20.1)			1.00	
Yes	18 (**75.0**)	**2.824**	**0.000**	**16.84**	**5.18–54.79**
Low social support					
No	24 (14.6)			1.00	
Yes	40 (**44.9**)	**1.081**	**0.005**	**2.95**	**1.39–6.29**
Notable intimate relationship dissatisfaction					
No	28 (17.4)			1.00	
Yes (< 13.5)	36 (**39.1**)	**0.892**	**0.020**	**2.44**	**1.15–5.16**

Note: Bold means *p* < 0.05

Specific sources of women’s stress included emotional or mental stress from making or accepting the abortion decision (82.2%), physical discomfort (78.7%), concerns of potential complications from the abortion (34.8%), intimate relationship dissatisfaction (28.9%), and keeping the abortion a secret (25.3%). Only 15% of women cited accessing abortion services as a stressor.

### Depression and associated factors

The average PHQ-9 score was 6.96 ± 4.90 with the overall rate of depression (PHQ ≥ 10) being 22.5%. Women with depression tended to be highly educated (p < 0.05), non-local residents (p < 0.01), with low resilience (p < 0.01), low perceived social support (p < 0.01), relationship dissatisfaction (p < 0.01), or experiencing high perceived stress (p < 0.01) (Table 3). The regression model revealed that depression was associated with high education level, non-local status, and high perceived stress (see supplementary file for detailed results). The adjusted odds ratio of factors contributing to depression were high perceived stress [aOR = 19.00, 95% CI 7.67–47.09] compared to women with normal or low perceived stress; having more than 12 years of education [aOR = 12.28, 95% CI 1.24–121.20] compared with less educated women; and non-local residents [aOR = 3.38, 95% CI 1.37–8.32] compared with local residents (Additional file 1: Table S1).

**Table 3:** Probable depression (PHQ ≥ 10) rates stratified by participant characteristics

Variables	Probable depression n (%)	F/χ^2^ (p value)
Demographic information		
Age		4.41 (0.110)
≤ 24	10 (27.0)	
25–34	30 (27.3)	
≥ 35	17 (16.0)	
Marital status		**3.24 (0.072)**
Unmarried	26 (28.9)	
Legally married	31 (19.0)	
Education		**5.12 (0.024)**
≤ 12 years	1 (4.2)	
> 12 years	56 (24.5)	
Religion		0.00 (1.000)
Atheist	57 (22.7)	
Others	0 (0)	
Monthly income (RMB)		1.99 (0.369)
< 1000	1 (10.0)	
1000–10,000	25 (20.3)	
> 10,000	31 (25.8)	
Household registration		**16.24 (0.000)**
Local	15 (11.9)	
Non-local	42 (33.1)	
Pre-existing physical condition		0.00 (1.000)
Yes	2 (25.0)	
No	55 (22.4)	
Reproductive history		
Parity		**5.07 (0.079)**
1	23 (29.9)	
2	16 (24.6)	
≥ 3	18 (16.2)	
Previous abortion		2.20 (0.138)
Yes	21 (18.3)	
No	36 (26.1)	
Existing child (ren)		2.34 (0.135)
No	32 (26.7)	
Yes	25 (18.8)	
Current status		
Gestation weeks		0.20 (0.650)
< 9 weeks	48 (23.1)	
≥ 9–12	9 (20.0)	
Pregnancy intention		2.36 (0.125)
Unintended	53 (24.1)	
Intended	4 (12.1)	
Abortion reason		0.56 (0.756)
Health-related reasons	19 (20.4)	
Social-related reasons	33 (23.2)	
Both	5 (27.8)	
Opinion about abortion		2.43 (0.119)
Pro-choice	48 (21.1)	
Situationist or pro-life	9 (34.6)	
Parenthood plan		3.63 (0.163)
Yes	11 (31.4)	
Current no, but yes in 2 years	19 (32.2)	
No	34 (21.4)	
Contraception		0.00 (0.972)
Yes	47 (22.5)	
No	10 (22.7)	
Domestic violence		**0.00 (1.000)**
No	55 (22.4)	
Yes	2 (28.6)	
Notable intimate relationship dissatisfaction		**6.70 (0.10)**
Yes	29 (31.5)	
No	28 (17.4)	
Low social support		**7.95 (0.005)**
Yes	29 (32.6)	
No	28 (17.1)	
Low resilience		**8.25 (0.004)**
Yes	11 (45.8)	
No	46 (20.1)	
High perceived stress		**84.67 (0.000)**
Yes	41 (64.1)	
No	16 (8.5)	

Note: Bold means *p* < 0.05

## Discussion

This study is one of the first to reveal high rates of perceived stress and depression among women seeking a first-trimester abortion in China. There was a consistent relationship between stress and depression. Women reported a relatively high level of stress compared to recent studies with the general public in China [17, 34]. Similarly, depression symptoms were higher than recent research with the non-infected Chinese population during the COVID-19 pandemic [34, 35]. These differences may be due to the psychological and social challenges arising from having an unwanted pregnancy and the abortion process. An evidence-based review conducted by the UK National Collaborating Centre for Mental Health (NCCMH) revealed an unwanted pregnancy and its resolution (abortion or give birth) often challenge women’s emotional wellbeing (contributing to negative emotions, stress, and mental disorders) [5].

The high rates of stress and depression among women seeking an abortion may also be affected by the influence of gender on coping [36]. For example, when facing similar stressful life events, women compared to men, tend to feel greater perceptions of threat, less control, and less social support [37]. Similarly, coping behaviours of women tend to be more cautious and avoidance focused, while men use emotional inhibition and detachment [36]. Women are also more likely to be dissatisfied with their intimate relationship than men [38], or experience more disadvantage as a migrant than their male counterparts [39]. These findings indicate the necessity for further investment in evaluating and promoting the mental health of women when seeking abortion, particularly among those with high-risk factors.

We found perceptions of high stress were associated with low resilience, not using contraceptives, low social support, non-local status, intimate relationship dissatisfaction, or pro-life attitudes towards abortion. Depression was associated with high perceived stress, high levels of education, and non-local status. These risk factors are congruent with a report on mental health and abortion by the APA Task Force [4]. Similar environmental factors (e.g., problematic intimate relationships, low social support, or exposure to other stressful life events) and personal characteristics (e.g., low resilience low self-efficacy, or problematic health behaviours) were identified [4]. Previous intervention studies suggest that women’s resilience [40], use of contraceptives [41], perceptions of social support [42], as well as intimate relationship satisfaction [38] are modifiable. For example, a systematic review and meta-analysis found cognitive behavioral therapy and interventions using mindfulness techniques can effectively improve individual resilience [40]. Another systematic review concluded that pre-abortion counselling was effective in promoting contraception use if accompanied by the provision of contraceptive methods [41]. Couples counselling or male partner involvement were also helpful [41].

The current study identified factors that are less amenable to change (like being a migrant, or holding pro-life attitudes towards abortion), but are meaningful to clinicians when assessing women’s vulnerability in order to provide comprehensive evaluation and timely support to women in high-risk groups [4].

In our study, only 2.8% of women reported experiencing violence in the preceding year, which was distinctly lower than rates (30–62%) reported in previous studies using similar brief self-report DV screening tools with women seeking an abortion [43, 44]. However, our study revealed a high level of intimate relationship dissatisfaction (31.5%) which is similar to existing studies [45]. Previous studies reveal that DV is closely related to relationship dissatisfaction [46, 47]. The observed unexpected disagreement between low DV experience and high relationship dissatisfaction in our study suggests the possibility of DV underreporting. It is possible that asking straightforward DV questions was insensitive or culturally inappropriate to detecting abuse among Chinese women seeking an abortion. Developing a trusting relationship with a known care provider is more likely to encourage disclosure from women about relationship violence.

### Limitations

Our findings should be interpreted in light of several limitations. Participating women were all from one Chinese metropolitan abortion clinic and most were atheists. The recruited sample may therefore not be representative of women in the wider community or women who practice their religious faith. The sensitive nature of studying the psychological status of Chinese women seeking a first-trimester induced abortion may have contributed to the under-reporting of some indicators like DV experience or abortion history.

Importantly, the study was conducted during the COVID-19 pandemic, even though only a small number of COVID-19 cases existed in Beijing during the survey period [48], and study participants were limited to those who were not infected or did not have a COVID-19 infection history. The enforcement of COVID related measures such as closing borders or mandatory quarantine could potentially impact women’s daily activities [49], the support resources at their disposal [50], as well as their actual abortion experience and associated psychological responses [51]. To determine the generalizability of our findings, we subsequently compared demographic and reproductive characteristics of women included in this study to a national cross-sectional study of 79,954 women in China who had an induced abortion in 2013 (pre-COVID). The analysis revealed that women aged 25–34 years represented 53.1% of the pre-COVID group versus 43.5% among this study group (0.01 < p < 0.05). Marital status was similar (68.2% in the pre-COVID group vs 64.4%, p > 0.05) as were the proportion of women who had children (57.3% pre-COVID vs 52.6%, p > 0.05) [52]. The mean gestational age of participating women was 43.7 ± 6.2 days ~ 59.0 ± 14.5 days in previously published studies [53–55], compared to 51 ± 7.3 days in the current study. Variation in the characteristics of women accessing abortion services pre- and post- COVID was not observed from this comparison. Future studies should, however, specifically investigate the impact of COVID on women’s abortion seeking behavior as well as their abortion experience.

## Conclusions

This study found a high prevalence of perceived stress and depression among Chinese women seeking a first-trimester induced abortion. Related risk factors of high perceived stress were low resilience, not-using contraceptives, low social support, non-local status, intimate relationship dissatisfaction, and holding pro-life attitudes towards abortion. High perceived stress, high education level, and non-local status were consistently associated with depression. Results reveal the necessity for evaluating the mental health of women seeking an abortion, especially those presenting with high-risk factors. Pre-and post- abortion counselling could be beneficial for all women seeking termination of their unwanted/unintended pregnancies. The identified risk factors can be used in screening high-risk groups and promoting the implementation of targeted interventions in abortion services to improve the psychological wellbeing of women.

## Supplementary Information

**Additional file 1**: Table S1 Factors associated with notable depression (PHQ ≥ 10) from binary logistic regression analysis.

## Data Availability

Research data are only available on request from the corresponding author due to ethical restrictions and policy concerns.

## Abbreviations

APA: American Psychological Association
BRS: Brief resilience scale
COVID-19: Corona virus disease in 2019
CSI-4: Couples satisfaction index (4-item)
DV: Domestic violence
DVST: Domestic violence screening tool
FPRHC: Family Planning and Reproductive Health Centre
IPV: Intimate partner violence
MOSSS-5: Medical outcomes study social support scale
NCCMH: National Collaborating Centre for Mental Health
OR: Odds ratio
PHQ: Patient health questionnaire
PSS: Perceived stress scale-10
QR code: Quick response code
SPSS: Statistical package for the social sciences
TFMHA: Task Force on Mental Health and Abortion
WHO: World Health Organization
