# Supplementary material for: Prevalence of stress and depression and associated factors among women seeking a first-trimester induced abortion in China: a cross-sectional study
Source: Reprod Health. 2022 Mar 9;19:64. doi: 10.1186/s12978-022-01366-1 (PMC8906361; doi:10.1186/s12978-022-01366-1)
Supplement: Supplementary file 1 — Additional file 1: Table S1 Factors associated with notable depression (PHQ ≥ 10) from binary logistic regression analysis. [file 12978_2022_1366_MOESM1_ESM.docx]

Additional file 1

Table S1 Factors associated with notable depression (PHQ≥10) from binary logistic regression analysis

| Variable | Notable depression rate (%) | N | B-coefficient | P-value | aOR | 95% CI |
| --- | --- | --- | --- | --- | --- | --- |
| Marital status |  |  |  |  |  |  |
| Unmarried | 28.9 | 26 |  |  | 1.00 |  |
| Legally Married | 19.0 | 31 | -0.526 | 0.318 | 0.59 | 0.21-1.66 |
| Education |  |  |  |  |  |  |
| ≤12 years | 4.2 | 1 |  |  |  |  |
| **>12 years** | **24.5** | **56** | **2.508** | **0.032** | **12.28** | **1.24-121.20** |
| Residence |  |  |  |  |  |  |
| Local | 11.9 | 15 |  |  | 1.00 |  |
| **Non-local** | **33.1** | **42** | **1.217** | **0.008** | **3.38** | **1.37-8.32** |
| Parity |  |  |  | 0.264 |  |  |
| 1 | 29.9 | 23 |  |  | 1.00 |  |
| 2 | 24.6 | 16 | -0.561 | 0.385 | 0.57 | 0.16-2.02 |
| ≥3 | 16.2 | 18 | 0.394 | 0.479 | 1.48 | 0.50-4.42 |
| Low Resilience |  |  |  |  |  |  |
| No | 20.1 | 46 |  |  | 1.00 |  |
| Yes | 45.8 | 11 | 0.245 | 0.690 | 1.28 | 0.38-4.26 |
| Low Social support |  |  |  |  |  |  |
| No | 17.1 | 28 |  |  | 1.00 |  |
| Yes | 32.6 | 29 | 0.008 | 0.986 | 1.01 | 0.41-2.50 |
| Notable Intimate relationship dissatisfaction |  |  |  |  |  |  |
| No | 17.4 | 28 |  |  | 1.00 |  |
| Yes (<13.5) | 31.5 | 29 | 0.313 | 0.470 | 1.37 | 0.59-3.20 |
| Perceived stress |  |  |  |  |  |  |
| Low | 8.5 | 16 |  |  | 1.00 |  |
| **High** | **64.1** | **41** | **2.945** | **0.000** | **19.00** | **7.67-47.09** |
